# Comprehensive Analysis of the Expression and Prognosis Value of Chromobox Family Members in Clear Cell Renal Cell Carcinoma

**DOI:** 10.3389/fonc.2021.700528

**Published:** 2021-07-28

**Authors:** Yuanyuan Zhu, Zhangya Pu, Zhenfen Li, Ying Lin, Ning Li, Fang Peng

**Affiliations:** ^1^Department of Blood Transfusion, Xiangya Hospital, Central South University, Changsha, China; ^2^National Health Commission (NHC) Key Laboratory of Cancer Proteomics, Xiangya Hospital, Central South University, Changsha, China; ^3^National Clinical Research Center for Geriatric Disorders, Xiangya Hospital, Central South University, Changsha, China; ^4^Department of Infectious Diseases and Hunan Key Laboratory of Viral Hepatitis, Xiangya Hospital, Central South University, Changsha, China; ^5^Department of Nuclear Medicine, Xiangya Hospital, Central South University, Changsha, China

**Keywords:** chromobox, biomarker, prognosis, bioinformatics analysis, clear cell renal cell carcinoma

## Abstract

Clear cell renal cell carcinoma (ccRCC) accounts for 80% of all renal cancers and has a poor prognosis. Chromobox (CBX) family protein expression has been reported in a variety of human malignancies, but the roles of CBXs in ccRCC remain unclear. In this study, by using ONCOMINE, UALCAN, GEPIA, Kaplan-Meier Plotter, cBioPortal, and TIMER, we found the transcriptional levels of CBX3 and CBX4 in ccRCC tissues were significantly higher than those in normal kidney tissues, whereas the transcriptional levels of CBX1, CBX5, CBX6, and CBX7 were significantly reduced in ccRCC tissues. The promoters of CBX2, CBX3, CBX4, CBX5, CBX6, CBX7, and CBX8 were hypermethylated, whereas the CBX1 promoter was hypomethylated in ccRCC. The expression of CBX1, CBX3, CBX4, CBX5, CBX6, and CBX7 was significantly associated with clinicopathological parameters in ccRCC patients. ccRCC patients with high expression levels of CBX3, CBX4, and CBX8 and low expression levels of CBX1, CBX5, CBX6, and CBX7 showed a strong association with poor overall survival. Genetic alterations in CBXs were correlated with poor overall survival and disease-free survival in patients with ccRCC. Moreover, we found significant associations between the expression of CBXs and infiltration of immune cells (B cells, CD8+ T cells, CD4+ T cells, macrophages, neutrophils, and dendritic cells). Our results provide novel insights into the development of CBX-based biomarkers and therapeutic targets for ccRCC.

## Introduction

Renal cell carcinoma (RCC) is the most prevalent urological cancer with a steady increase in mortality and morbidity in recent years (1). Clear cell renal cell carcinoma (ccRCC), the most prevalent subtype of RCC, accounts for more than 75% of the RCC cases (2). Despite recent advances in the detection and treatment of RCC, the clinical outcomes of patients with metastatic RCC remain poor. The 5-year survival rate is less than 10% (3). In the last few decades, several studies have been carried out on the pathogenesis of ccRCC, but the detailed molecular events of ccRCC occurrence and development are not yet fully understood. Therefore, it is necessary to identify more effective therapeutic targets and prognostic markers for ccRCC.

Chromobox (CBX) family members are typical components of the polycomb repressive complex (PRC) that epigenetically modifies chromatin and participates in multiple physiological processes, such as differentiation, senescence, and DNA repair (4). CBX family proteins can be divided into two types: heterochromatin protein 1 (HP1) group (containing CBX1, CBX3, and CBX5), with an N-terminal chromodomain, and polycomb (Pc) group (containing CBX2, CBX4, CBX6, CBX7, and CBX8), with a conserved N-terminal chromodomain and a C-terminal Pc repressor box (5). Evidence has demonstrated that CBXs play complex roles in human tumors. CBXs can exert both anti-tumor and pro-tumor activities depending on the tumor type and cellular context. For example, studies have shown that CBX4 can promote angiogenesis of liver tumors through HIF-α sumoylation, which is a negative factor for the prognosis of patients with hepatocellular carcinoma (6). CBX4 also inhibits the expression of Runx2 by recruiting HDAC3, thereby inhibiting tumor metastasis in colorectal cancer (7). CBX7 shows inhibitory activity in thyroid, colon, and lung cancer (8–10), while playing an oncogenic role in gastric cancer (11). In ccRCC, CBX4 transcriptionally suppresses KLF6 by interacting with HDAC1 to exert oncogenic activities (12). However, the roles of other members of the CBX family in the development and progression of ccRCC remain unknown.

In this study, we aimed to analyze the expression and clinical relevance of CBX family members in ccRCC based on online public databases. We analyzed expression patterns, clinicopathological parameters, prognostic values, genetic alterations, and immune cell infiltration. Our findings indicate that CBXs may have complex and distinct functions in ccRCC and serve as potential therapeutic targets for the clinical intervention of ccRCC in the future.

## Materials and Methods

### UALCAN

UALCAN (http://ualcan.path.uab.edu) is a comprehensive and interactive website that provides cancer data based on TCGA database (13). In this study, data on the mRNA expression and promoter methylation levels as well as the association of the transcriptional expression of CBXs with clinicopathologic parameters in ccRCC patients were obtained using UALCAN. The Student’s t-test was used to compare differences, and statistical significance was set at *P* < 0.05.

### ONCOMINE

ONCOMINE (www.oncomine.org) is an online cancer database used for powerful genome-wide expression analysis (14). Data on significant changes in the transcription levels of CBXs between ccRCC and normal kidney tissues were obtained from the ONCOMINE database. The cutoffs of the *P* value and fold change were 0.01 and 1.5, respectively.

### Human Protein Atlas

The Human Protein Atlas (https://www.proteinatlas.org) is a website that provides the distribution and expression of protein in normal human tissues, tumor tissues, cell lines, and blood cells by immunohistochemistry (15). In this study, immunohistochemistry images were used to directly compare the protein expression of different CBX family members in human normal and ccRCC tissues.

### GEPIA

GEPIA (http://gepia.cancer-pku.cn/index.html) is a newly developed website for interactive gene expression profiling analysis (16). In this study, disease-free survival (DFS) analysis of CBXs in ccRCC patients was performed using a Kaplan-Meier curve, with hazard ratios (HRs) and 95% confidence intervals (CIs). In addition, the relative expression levels of CBXs in ccRCC tissues were analyzed using GEPIA.

### Kaplan-Meier Plotter

The prognostic value of CBXs in patients with ccRCC was analyzed using the Kaplan-Meier plotter (http://kmplot.com/analysis/) (17). Based on CBX expression levels, ccRCC patients were divided into two groups and the data were assessed using a Kaplan-Meier survival plot. Data were considered statistically significant at *P* < 0.05.

### cBioPortal

cBioPortal (http://www.cbioportal.org/) is a comprehensive web resource that can be used to explore multidimensional cancer genomics data (18). We obtained the genetic alterations of CBXs in ccRCC from cBioPortal (TCGA, PanCancer Atlas). mRNA expression z-scores (RNA Seq V2 RSEM) were obtained using a z-score threshold of ±1.8. Moreover, genetic alterations in CBXs and their relationships with overall survival (OS) and the disease-free survival (DFS) in patients with ccRCC were analyzed using Kaplan-Meier curves. Statistical significance was set at *P* < 0.05.

### TIMER

Timer (https://cistrome.shinyapps.io/timer/) is a website for the comprehensive analysis of tumor-infiltrating immune cells and their impact on clinical outcomes (19). In this study, the “Gene module” was used to explore the correlation between CBX expression and the abundance of immune infiltrates, and the “Survival module” was used to analyze the association between the infiltration of immune cells and CBXs with the clinical outcome of ccRCC patients.

### GSEA Analysis

Gene Set Enrichment Analysis (GSEA) is a computational method that determines whether a set of *priori* defined genes shows statistically significant, concordant differences between two biological states. GSEA was obtained from the Broad Institute (http://software.broadinstitute.org/gsea/index.jsp) (20). The cut-off values were predefined as *P* < 0.05 and FDR < 0.25.

### Cell Culture

The ccRCC cell lines (786-O and 769-P) were purchased from Chinese Academy of Sciences Cell Bank (Shanghai, China) and HK2 cell was provided by NHC Key Laboratory of Cancer Proteomics, Xiangya Hospital, Central South University (Changsha, China). 786-O and 769-P cells were cultured in RPMI-1640 medium (Gibco, USA), and HK2 cell was cultured in Dulbecco’s modified Eagle’s medium (DMEM, Gibco, USA), both were supplemented with 10% fetal bovine serum (Gibco, USA) and 1% penicillin-streptomycin (Gibco, USA) in a humidified incubator at 37°C containing 5% CO_2_.

### RNA Extraction and qRT-PCR

Total RNA was extracted from cell using Trizol reagent (Takara, Dalian, China) and the RNA quality and concentration were assessed using a NanoDrop™ 1000 Spectrophotometer (Thermo Fisher Scientific, USA). The OD260/OD280 ratios were 1.8-2.0, and the OD260/OD230 ratios were 2.0-2.2. Then the complementary DNA was synthesized by reverse transcription reaction using the TaqMan MicroRNA Reverse Transcription Kit (Applied Biosystems, USA). The qRT-PCR was performed with miScript SYBR Green PCR kit (Qiagen) using an ABI7500 instrument (Applied Biosystems). The PCR conditions were 95°C for 30 sec, 45 cycles at 94°C for 15 sec and one step at 60°C for 31 sec, and 55°C for 30 sec. ACTB was used as a normalizer for RNA quantification. The data were analyzed using the 2^–ΔΔCt^ method.

### Western Blotting

Cells were lysed by lysis buffer (150 mM NaCl, 50 mM Tris-HCl, pH7.5) supplemented with inhibitors (1 mM phenylmethylsulphonyl fluoride, 1 mg/ml aprotinin, 1 mg/ml leupeptin, 1 mg/ml pepstatin, 1 mM Na_3_VO_4_, 1 mM NaF). Then cell lysates were centrifuged at 4°C, 14000 g for 20 minutes to remove cell debris. Bicinchonininc acid (BCA) assay kit (BCA Assay Reagent, Pierce Chemical, America) was used to determine the protein content. For western blotting, protein samples were separated by SDS-PAGE and transferred to the polyvinylidene fluoride (PVDF) membrane. Using primary antibodies incubated the target proteins overnight at 4°C, then the membranes were incubated with corresponding secondary antibodies, including anti-rabbit and anti-mouse horseradish peroxidase (HRP)-linked IgG (Proteintech,1:5000) for 2 hours. The working dilution of primary antibodies were: CBX3: 1:1000 (Immunoway, YM3741); CBX6: 1:1000 (Immunoway, YT0699); CBX7: 1:1000 (Sangon Biotech, D262675); α-Tubulin: 1:5000 (Proteintech, 66031-1-Ig).

### Immunohistochemistry

Tissue microarrays containing 9 human ccRCC tissues and paired adjacent normal kidney tissues were purchased from Avilabio (DC-Kid11008). The experimental operation was conducted according to the protocol of the immunohistochemical staining kit (ZSGB-BIO, PV-9000). The working dilution of primary antibodies were: CBX3: 1:200 (Immunoway, YM3741); CBX6: 1:200 (Immunoway, YT0699); CBX7: 1:50 (Sangon Biotech, D262675). The research was approved by the local ethics committee. To quantify the level of protein expression, five random fields in each tissue sample were photographed with a microscope and analyzed by two experienced pathologists. The expression levels were scored as proportion of immunopositive staining area (0 for ≤5% positive cells, 1 for 6–25%, 2 for 26–50%, 3 for 51–75%, and 4 for ≥76%) multiplied by intensity of staining (0 for negative staining, 1 for weak staining, 2 for moderate staining, and 3 for strong staining).

### Statistical Analysis

Data were presented as the means ± standard error of the mean (SEM). Statistical analyses were done using SPSS. Student’s t-test were used for comparison between groups. *P* < 0.05 were considered statistically significant.

## Results

### Expression Levels and Promoter Methylation Status of CBXs in Patients With ccRCC

**Figure 1:**
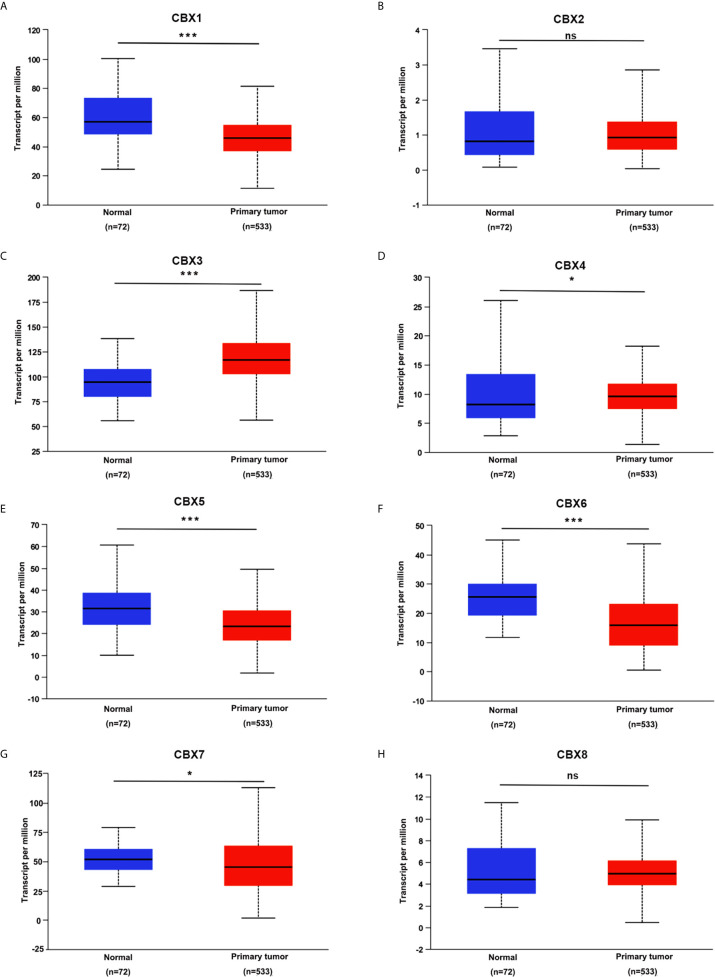
The transcription levels of CBXs in ccRCC (UALCAN). The transcription levels of CBX3 and CBX4 in ccRCC tissues were significantly elevated compared with normal kidney tissues **(C, D)**, while the transcriptional levels of CBX1, CBX5, CBX6, CBX7 were significantly reduced in ccRCC tissues **(A, E–G)**. The CBX2 and CBX8 were expressed at similar levels in ccRCC tissues and normal kidney tissues **(B, H)**. (*p<0.05, ***p<0.001, ns: no significance).

**Figure 2:**
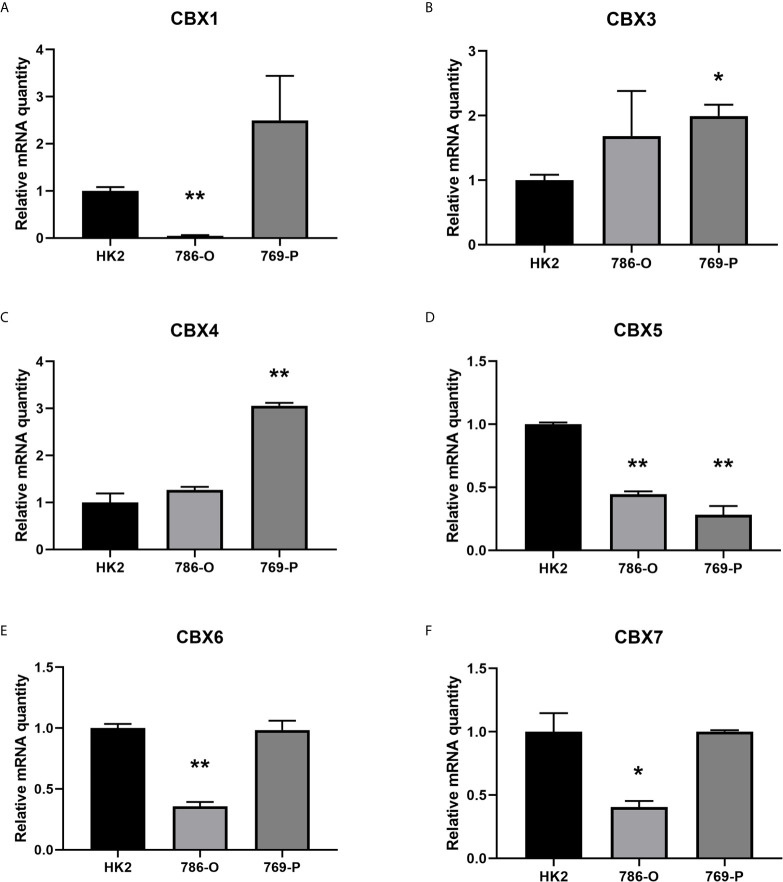
The mRNA levels of CBXs in normal kidney cell and ccRCC cells (qRT-PCR). The mRNA levels of CBX3 and CBX4 in ccRCC cells were significantly elevated compared with normal kidney cells **(B, C)**, while the mRNA levels of CBX1, CBX5, CBX6, CBX7 were significantly reduced in ccRCC cells **(A, D–F)**. (*p<0.05, **p<0.01).

We firstly analyzed the mRNA expression of different CBX family members in ccRCC patients using the UALCAN and ONCOMINE databases. As shown in **Figure 1**, the transcriptional levels of CBX3 and CBX4 in ccRCC tissues were significantly higher than those in normal kidney tissues, whereas the transcriptional levels of CBX1, CBX5, CBX6, and CBX7 were significantly reduced in ccRCC tissues. CBX2 and CBX8 were expressed at similar levels in ccRCC and normal kidney tissues. Next, the transcription levels of the CBX family members were measured using the ONCOMINE database. The expression levels of CBX1, CBX3, CBX4, CBX5, and CBX7 also showed significant changes in ccRCC **(** **Table 1** **)**. And the mRNA expressions of CBXs in normal kidney cell HK2 and ccRCC cells 786-O and 769-P were analyzed using qRT-PCR. The transcriptional levels of CBX3 and CBX4 in ccRCC cells were significantly higher than those in normal kidney cell, whereas the transcriptional levels of CBX1, CBX5, CBX6, and CBX7 were significantly reduced in ccRCC cells **(** **Figure 2** **)**, which was consistent with the database. The abundance of CBX2 and CBX8 in both normal kidney cell and ccRCC cells were very low, and the qRT-PCR cycle number were more than 45.

**Table 1:** The significant changes of CBXs expression in transcription level between ccRCC and normal kidney tissues (Oncomine).

	Type	Fold change	*P*-value	t-test	References
CBX1	Clear Cell Renal Cell Carcinoma	2.307	1.49E-5	5.119	TCGA
CBX3	Hereditary Clear Cell Renal Cell Carcinoma	2.052	1.03E-13	12.781	(21)
	Non-Hereditary Clear Cell Renal Cell Carcinoma	1.882	1.43E-9	7.826	(21)
	Clear Cell Renal Cell Carcinoma	2.541	1.33E-14	11.784	(22)
	Clear Cell Renal Cell Carcinoma	3.134	1.93E-6	6.215	(22)
	Clear Cell Renal Cell Carcinoma	1.784	1.91E-6	6.560	(23)
	Clear Cell Renal Cell Carcinoma	2.296	6.36E-4	4.110	(24)
CBX4	Clear Cell Renal Cell Carcinoma	1.843	0.002	3.443	(24)
CBX5	Clear Cell Renal Cell Carcinoma	1.703	0.006	3.317	(25)
	Clear Cell Renal Cell Carcinoma	1.598	0.005	2.956	(24)
CBX7	Clear Cell Renal Cell Carcinoma	2.166	1.33E-4	4.012	(26)

**Figure 3:**
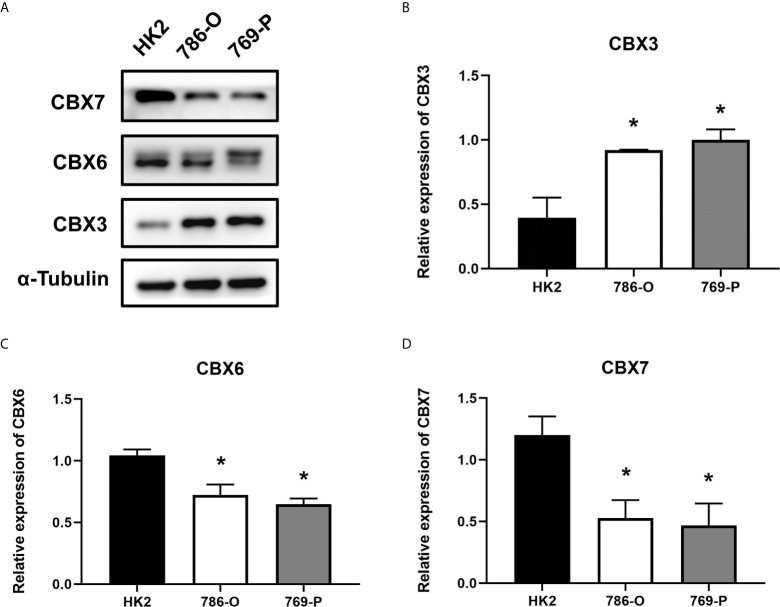
The protein expression levels of CBX3, CBX6 and CBX7 in normal kidney cell and ccRCC cells (Western blotting). CBX3 protein was much higher in ccRCC cell lines than that in HK2 cells, while CBX6 and CBX7 were significantly reduced in ccRCC cells. The representative western blotting image of CBX3, CBX6 and CBX7 in ccRCC cells and normal kidney cells **(A)**. The statistical results of western blotting of CBX3, CBX6 and CBX7 in ccRCC cells and normal kidney cells **(B–D)**. (*P<0.05).

**Figure 4:**
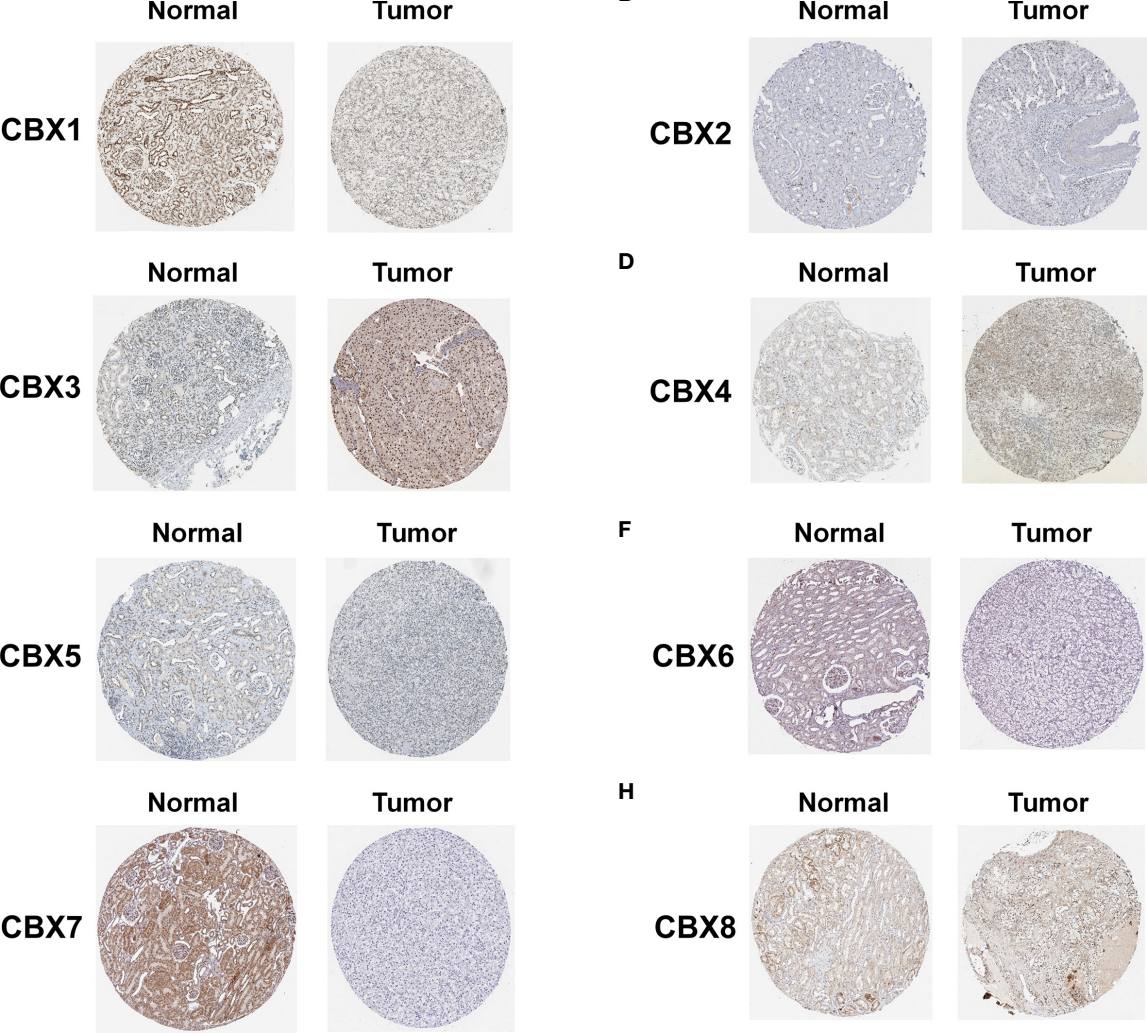
The protein expression of CBXs in normal kidney tissues and ccRCC tissues (Human Protein Atlas). The protein expression of CBX3 and CBX4 in ccRCC tissues were significantly elevated compared with normal kidney tissues **(C, D)**, while the protein expression of CBX1,CBX5, CBX6, CBX7 were significantly reduced in ccRCC tissues **(A, E–G)**. The CBX2 and CBX8 were expressed at similar levels in ccRCC tissues and normal kidney tissues **(B, H)**.

**Figure 5:**
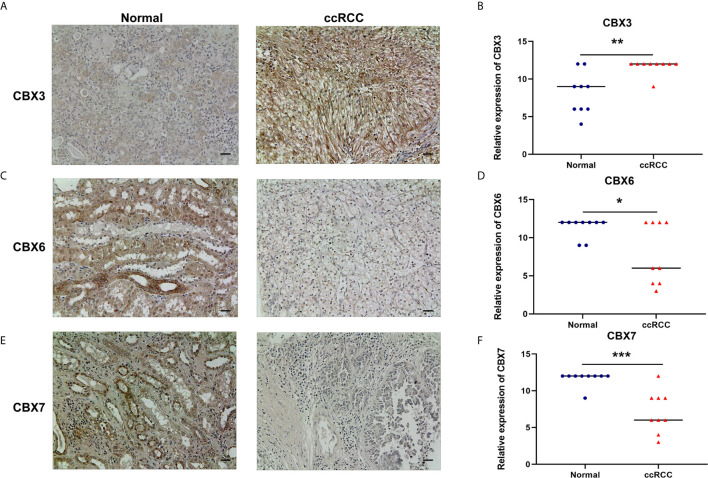
The protein expression levels of CBX3, CBX6 and CBX7 in ccRCC tissues and paired adjacent normal kidney tissues (Immunohistochemistry). CBX3 was overexpressed in ccRCC tissues compared to normal kidney tissues, while expression levels of CBX6 and CBX7 were reduced in ccRCC tissues. The representative immunohistochemistry image of CBX3, CBX6 and CBX7 in ccRCC tissues and normal kidney tissues **(A, C, E)**. The scatter plot of immunohistochemistry score **(B, D, F)**. (*P<0.05, **P<0.01, ***P<0.001).

**Figure 6:**
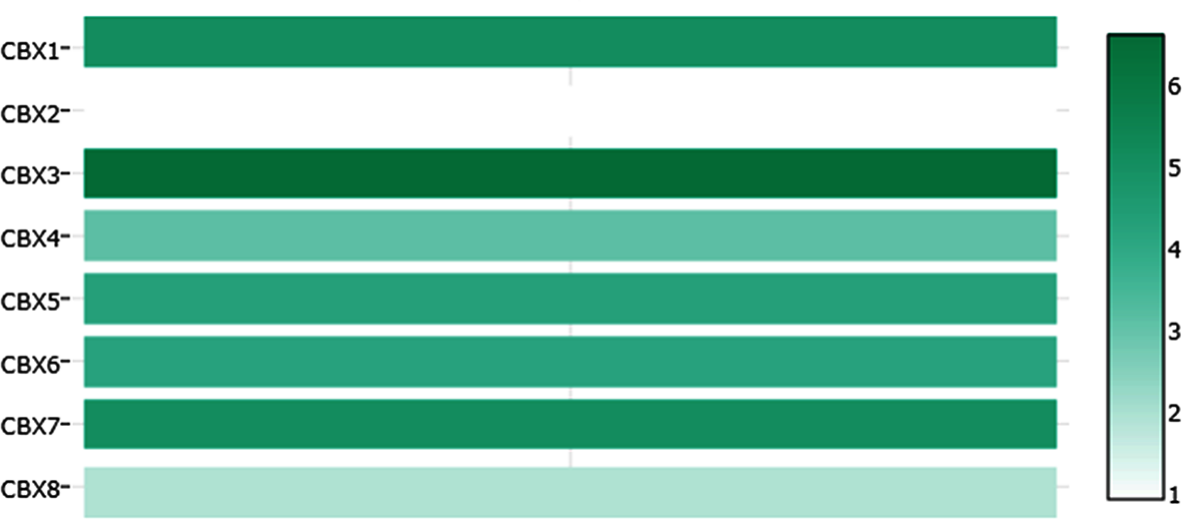
The relative expression levels of CBXs in ccRCC (GEPIA).

**Figure 7:**
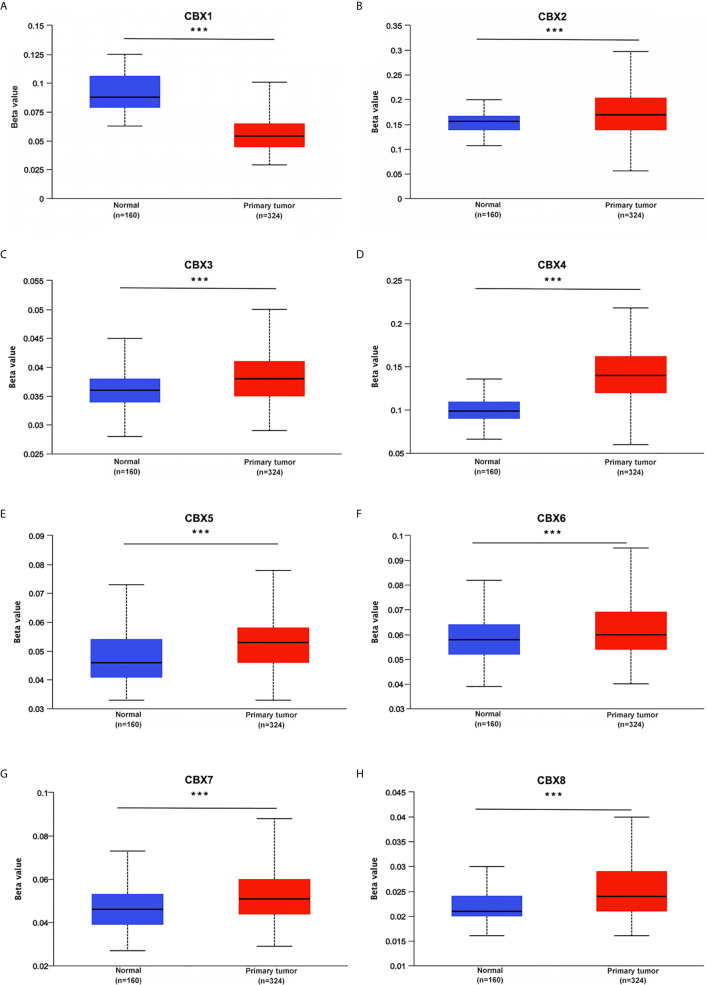
Promoter methylation status of CBXs in ccRCC (UALCAN). The promoters of CBX2, CBX3, CBX4, CBX5, CBX6, CBX7 and CBX8 were hypermethylated in ccRCC tissues **(B–H)**, while CBX1 promoter was hypomethylated in ccRCC tissues than that in normal kidney tissues **(A)**. (***P<0.001).

We also explored the protein expression of CBXs in ccRCC. Firstly, we examined the protein expression levels of CBX3, CBX5 and CBX6 in HK2, 786-O and 769-P cell lines. The results showed that CBX3 protein was much higher in ccRCC cell lines than that in HK2 cells, while CBX6 and CBX7 were significantly reduced in ccRCC cells (**Figure 3** **)**. And Human Protein Atlas data showed that low protein expressions of CBX3 and CBX4 were found in normal kidney tissues, while medium and high protein expressions of them were observed in ccRCC tissues **(** **Figures 4C, D** **)**. However, higher protein expressions of CBX1, CBX5, CBX6 and CBX7 were observed in normal kidney tissues than in ccRCC tissues **(** **Figures 4A, E–G** **)**. CBX2 protein was not expressed both in normal kidney tissues and ccRCC tissues **(** **Figure 4B** **)**. CBX8 protein was expressed at similar level in ccRCC and normal kidney tissues **(** **Figure 4H** **)**. Our own immunohistochemical results suggestd that CBX3 was overexpressed in ccRCC tissues, while expression levels of CBX6 and CBX7 were reduced in ccRCC tissues **(** **Figure 5** **)**, which were consistent with the database. We also compared the relative expression levels of CBXs in ccRCC tissues and found that the relative expression of CBX3 was the highest among all CBXs **(** **Figure 6** **)**. Moreover, given the important role of promoter methylation in the process of renal tumors, we used UALCAN to investigate the promoter methylation levels of CBXs in ccRCC and found that the promoters of CBX2, CBX3, CBX4, CBX5, CBX6, CBX7, and CBX8 were hypermethylated in ccRCC tissues, while the CBX1 promoter was hypomethylated in ccRCC tissues, compared to those in the normal kidney tissues **(** **Figure 7** **)**.

### The Relationship of mRNA Levels of CBXs With Clinicopathological Parameters in Patients With ccRCC

**Figure 8:**
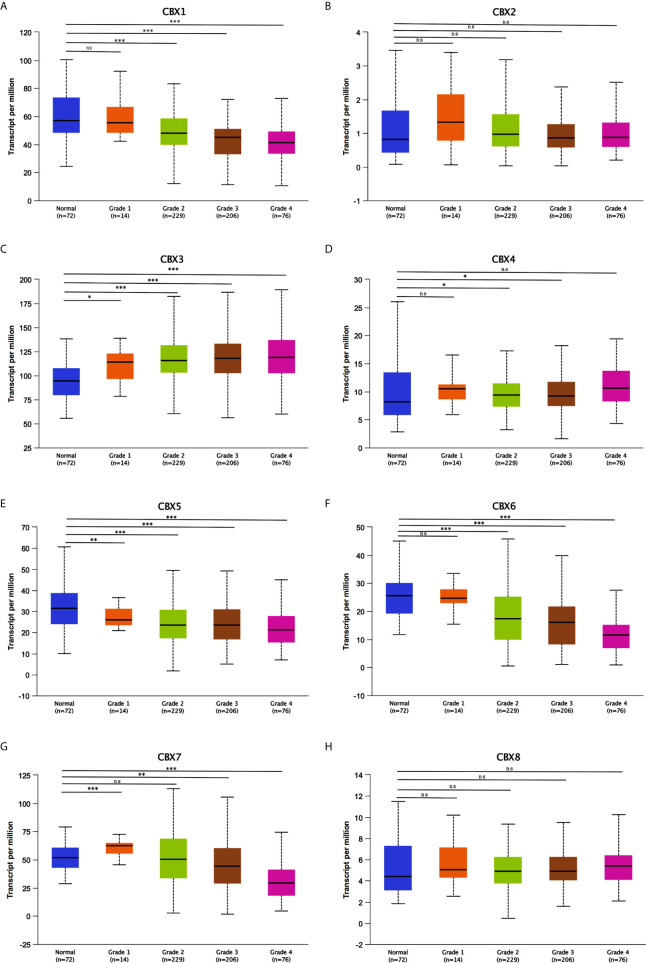
Association of mRNA expression of CBXs with tumor grades of ccRCC patients (UALCAN). ccRCC patients who were in more advanced tumor grades tended to express higher mRNA expression of CBX3 and CBX4 **(C, D)**, while lower mRNA expression of CBX1, CBX5, CBX6 and CBX7 **(A, E–G)**. CBX2 and CBX8 did not significantly differ in tumor grades **(B, H)**. (*P<0.05, **P<0.01, ***P<0.001, ns: no significance).

**Figure 9:**
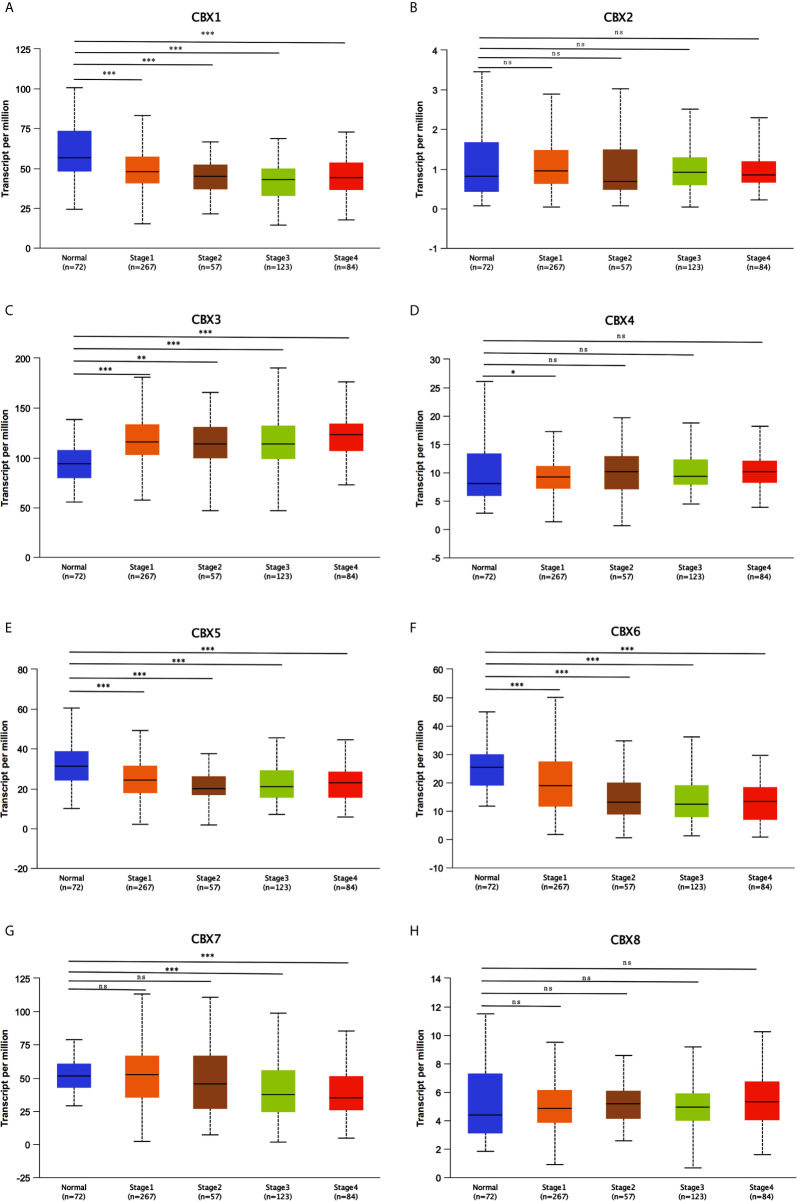
Correlation between CBXs expression and tumor stages in ccRCC patients (UALCAN). Low expression of CBX1, CBX5, CBX6, CBX7 **(A, E–G)**, and high expression of CBX3, CBX4 **(C, D)** were significantly correlated with advanced cancer stages. CBX2 and CBX8 did not significantly differ in tumor stages **(B, H)**. (*P<0.05, **P<0.01, ***P<0.001, ns, no significance).

After mRNA expression and promoter methylation were analyzed, we next explored the relationship between mRNA levels of CBXs and clinicopathological parameters in ccRCC patients using UALCAN; this included the patients’ cancer stages and tumor grades. The mRNA expression of CBXs was significantly correlated with cancer stage and tumor grade. As the tumor progressed, patients with more advanced tumor grades tended to express higher mRNA expression of CBX3 and CBX4 but lower mRNA expression of CBX1, CBX5, CBX6, and CBX7 **(** **Figure 8** **)**. In addition, as shown in **Figure 9**, low expression of CBX1, CBX5, CBX6, and CBX7 and high expression of CBX3 and CBX4 were significantly correlated with advanced cancer stages. CBX2 and CBX8 expression did not differ significantly with cancer stage or tumor grade. In conclusion, mRNA expression of CBX1, CBX3, CBX4, CBX5, CBX6, and CBX7 was significantly associated with clinicopathological parameters in ccRCC patients.

### The Prognostic Value of CBXs in Patients With ccRCC

**Figure 10:**
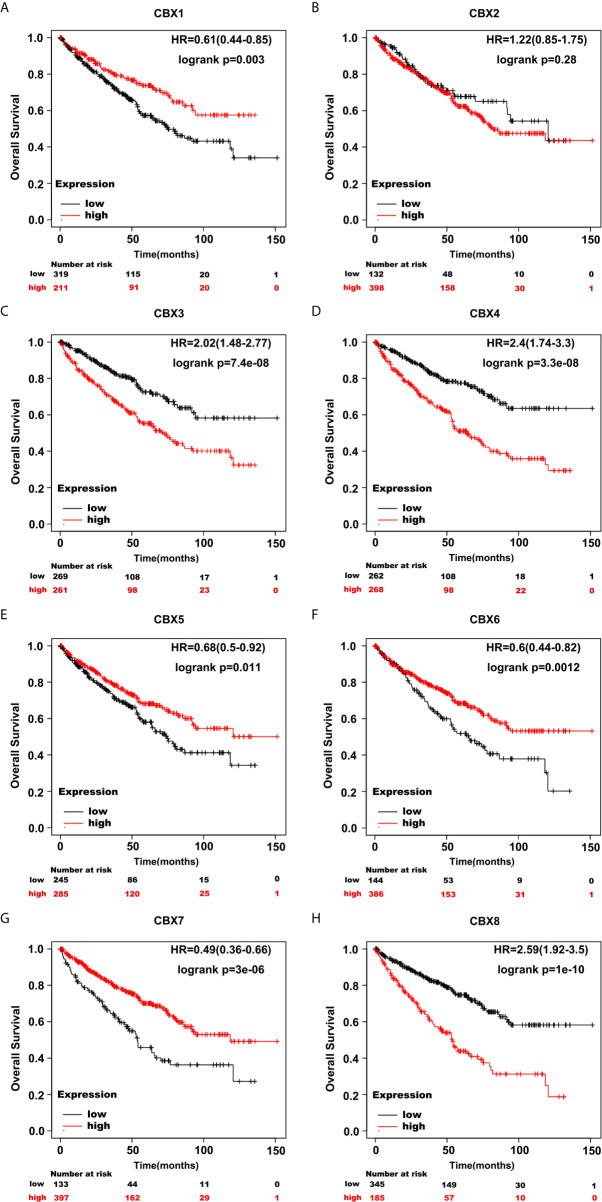
The prognostic value of CBXs in ccRCC patients in the OS curve (Kaplan-Meier Plotter). ccRCC patients with high expression levels of CBX3, CBX4, CBX8 **(C, D, H)**, and low expression levels of CBX1, CBX5, CBX6, CBX7 **(A, E–G)** were strongly associated with poor overall survival. However, CBX2 expression showed no correlation with overall survival in ccRCC patients **(B)**.

**Figure 11:**
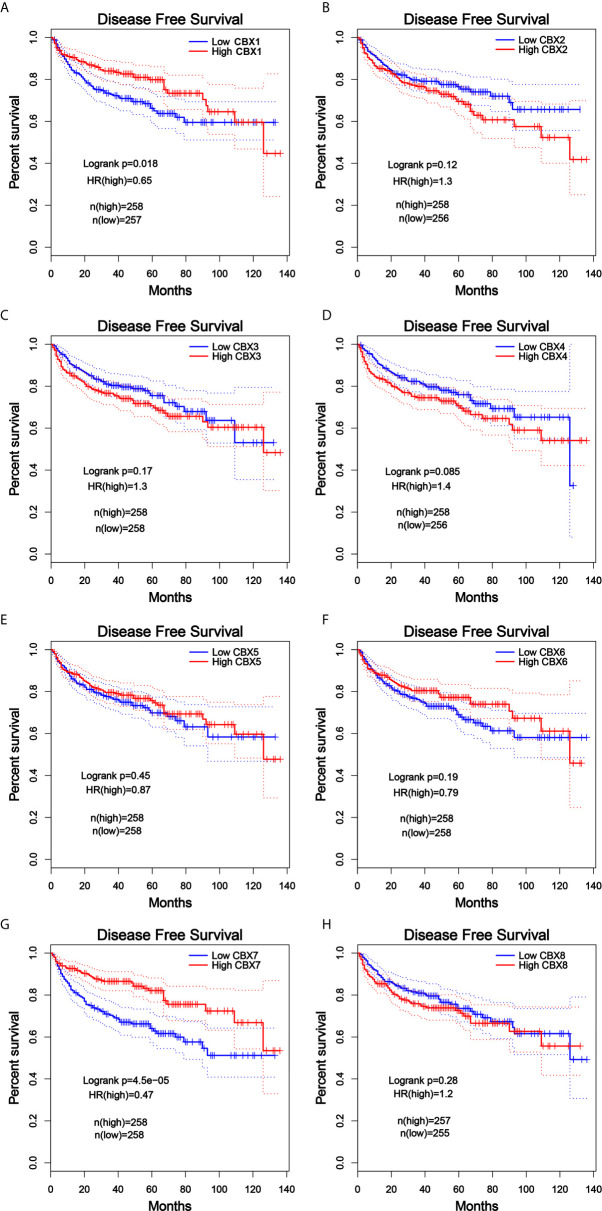
The prognostic value of different expressed CBXs in ccRCC patients in the DFS curve (GEPIA). Low expression levels of CBX1 and CBX7 were significantly correlated with poor disease-free survival in ccRCC patients **(A, G)**. CBX2, CBX3, CBX4, CBX5, CBX6 and CBX8 expression showed no correlation with disease free survival in ccRCC patients **(B–F, H)**.

Furthermore, we analyzed the prognostic value of CBXs in patients with ccRCC using the Kaplan-Meier plotter and GEPIA database. ccRCC patients with high expression levels of CBX3, CBX4, and CBX8, and low expression levels of CBX1, CBX5, CBX6, and CBX7 showed a strong association with poor OS **(** **Figure 10** **)**. The value of differentially expressed CBXs in DFS of ccRCC patients was also evaluated. The results showed that low expression levels of CBX1 and CBX7 were significantly correlated with poor DFS **(** **Figure 11** **)**. These results indicated that the expression levels of CBX1, CBX3, CBX4, CBX5, CBX6, CBX7, and CBX8 were significantly associated with ccRCC patient prognosis, and they might be exploited as useful biomarkers for predicting the survival of patients with ccRCC.

### Genetic Alteration of CBXs and Their Relationship With OS and DFS in Patients With ccRCC

**Figure 12:**
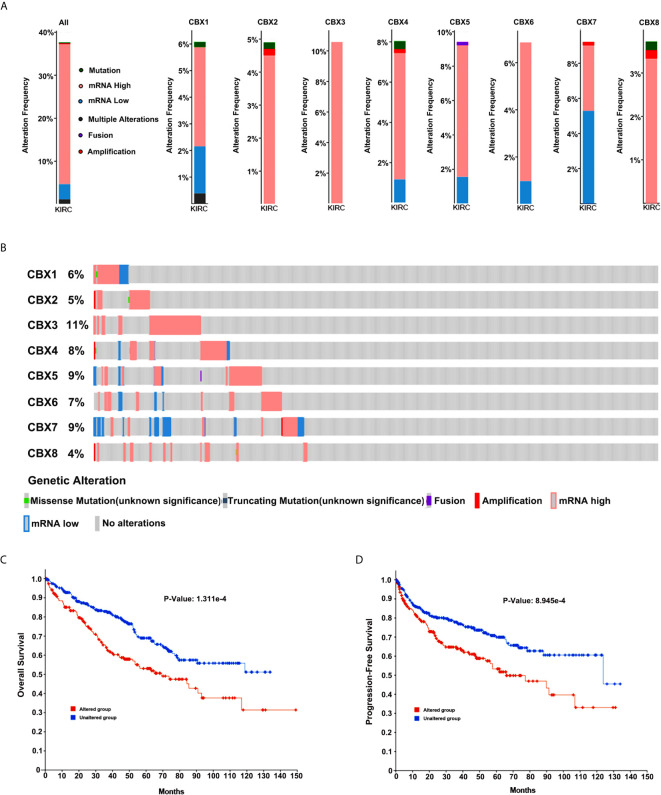
Genetic alteration in CBXs and their relationship with OS and DFS of ccRCC patients (cBioPortal). The genetic alterations rates of CBX1, CBX2, CBX3, CBX4, CBX5, CBX6, CBX7 and CBX8 **(A, B)**. Correlation of CBXs gene alterations with OS and DFS in patients with ccRCC **(C, D)**.

Next, we applied the cBioPortal online tool (TCGA, PanCancer Atlas) to investigate genetic alterations in CBXs and their relationship with OS and DFS in ccRCC. In the 510 sequenced ccRCC patients, genetic alterations were found in 192 patients, and the alteration rate was 38%. The OncoPrints included missense mutation, truncating mutation, fusion, deletion, amplification, as well as high and low levels of mRNA. The rates of genetic alterations of CBX1, CBX2, CBX3, CBX4, CBX5, CBX6, CBX7, and CBX8 were 6%, 5%, 11%, 8%, 9%, 7%, 9%, and 4%, respectively **(** **Figures 12A, B** **)**. Moreover, Kaplan-Meier plots and log-rank tests indicated that genetic alterations in CBXs were correlated with poor OS and DFS in patients with ccRCC **(** **Figures 12C, D** **)**

### Immune Cell Infiltration of CBXs in Patients With ccRCC

**Figure 13:**
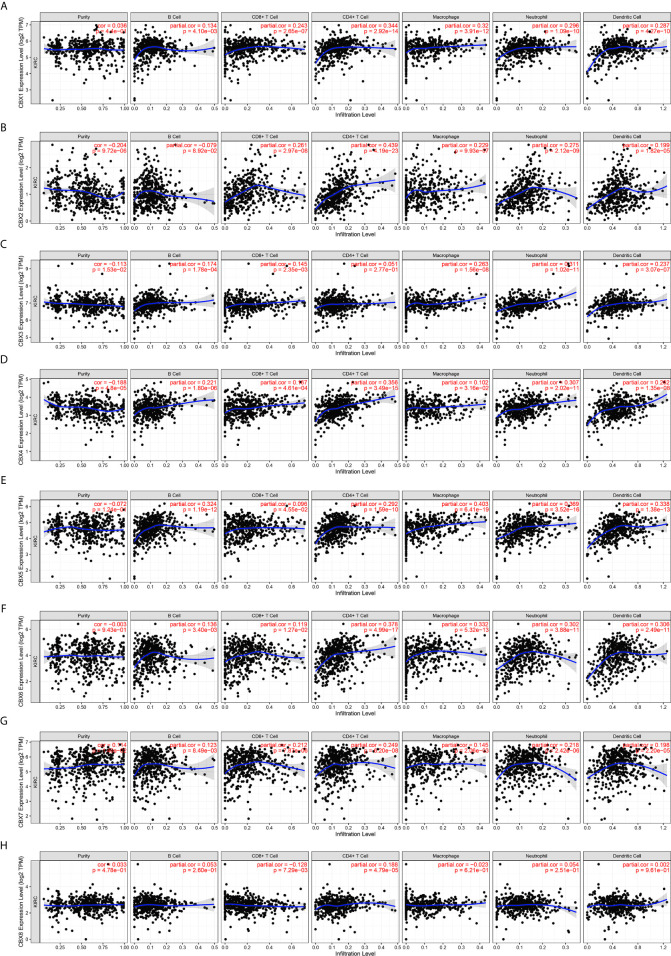
The correlation between differentially expressed CBXs and immune cells infiltration (TIMER). CBX1, CBX2, CBX4, CBX5, CBX6 and CBX7 expression was positively correlated with the infiltration of B cell, CD8+ T cells, CD4+ T cells, macrophage, neutrophils and dendritic cells **(A, B, D–G)**. And there was a positive correlation between CBX3 expression and the infiltration of B cell, CD8+ T cells, macrophage, neutrophils and dendritic cells **(C)**. The expression of CBX8 was positively associated with the infiltration of CD4+ T cells, and negatively associated with the infiltration of CD8+ T cells **(H)**.

Inflammatory responses and immune cell infiltration can affect the clinical outcomes of cancer patients. Therefore, we comprehensively analyzed the association between CBX expression levels and immune cell infiltration using the TIMER database. CBX1, CBX2, CBX4, CBX5, CBX6, and CBX7 expression was positively correlated with the infiltration of B cells, CD8+ T cells, CD4+ T cells, macrophages, neutrophils, and dendritic cells **(** **Figures 13A, B, D–G** **)**. There was a positive correlation between CBX3 expression and the infiltration of B cells, CD8+ T cells, macrophages, neutrophils, and dendritic cells **(** **Figure 13C** **)**. Similarly, the expression of CBX8 was positively associated with the infiltration of CD4+ T cells and negatively associated with the infiltration of CD8+ T cells **(** **Figure 13H** **)**. Moreover, we used the Cox proportional hazard model and found that CD8+ T cells, CBX4, CBX7, and CBX8 were significantly correlated with the clinical outcome of ccRCC patients after adjustment for confounding factors **(** **Table 2** **)**.

**Table 2:** The cox proportional hazard model of CBXs and six tumor-infiltrating immune cells in ccRCC (TIMER).

	coef	HR	95% CI_l	95% CI_u	*P*-value	sig
B_cell	-1.374	0.253	0.011	5.606	0.385	
CD8_Tcell	-1.884	0.152	0.028	0.818	0.028	*
CD4_Tcell	-1.320	0.267	0.015	4.839	0.372	
Macrophage	-1.452	0.234	0.026	2.133	0.198	
Neutrophil	3.374	29.189	0.699	1218.706	0.076	
Dendritic	1.467	4.335	0.747	25.147	0.102	
CBX1	0.177	1.194	0.759	1.876	0.443	
CBX2	-0.137	0.872	0.564	1.349	0.538	
CBX3	0.013	1.013	0.626	1.639	0.958	
CBX4	0.488	1.629	1.079	2.460	0.020	*
CBX5	-0.160	0.852	0.574	1.265	0.428	
CBX6	-0.302	0.739	0.543	1.006	0.055	
CBX7	-0.280	0.756	0.601	0.950	0.016	*
CBX8	0.515	1.673	1.204	2.326	0.002	**

*P < 0.05, **P < 0.01.

### GSEA Analysis of the Gene Expression Files in CBXs mRNA High Subgroup *Versus* Low Subgroup

We also used GSEA analysis to determine the gene expression files in CBX family mRNA high versus low. GSEA results showed that several gene sets that were significantly enriched in CBXs mRNA high subgroup compared to low subgroup, including Wnt signaling pathway, oxidative phosphorylation, tight junction, endocytosis, spliceosome. While proximal tubule bicarbonate reclamation, cytosolic DNA-sensing pathway and pathways in cancer were significantly enriched in CBXs mRNA low subgroup compared to high subgroup **(** **Supplementary Figure 1** **)**.

## Discussion

Epigenetic modification has been proven to be related to multiple physiological processes, including differentiation, senescence, and DNA repair (27–30). As important components of epigenetic regulation complexes, CBX family proteins can mediate the recruitment of PRC1 to chromatin and participate in the occurrence and progression of various tumors (31, 32). CBX family proteins function as both oncogenes and tumor suppressors, depending on the tumor type and cellular context. However, to date, the prognostic value and biological function of CBX family members in the progression of ccRCC remain unknown. In this study, we found that six out of eight CBX family members were differentially expressed in ccRCC, and seven CBX family members were significantly associated with the survival of ccRCC patients. In addition, we showed that the expression of six CBX family members was closely related to cancer stage and tumor grade in ccRCC. The expression of CBX family members was associated with the infiltration of six immune cells and influenced the outcome of ccRCC patients. Moreover, we found that promoter methylation status and genetic alteration might cause dysregulation of CBX family members in ccRCC. For most members of the CBX family, our study was the first to systematically analyze the their expression and prognostic significance in ccRCC.

In previous studies, overexpression of CBX1 has been found in many cancers such as hepatocellular carcinoma (33, 34), gastric cancer (35, 36), colorectal cancer (37), and pituitary cancer (38). In HCC, CBX1 functions as an oncogene by interacting with the transcription factor HMGA2 to activate the Wnt/β-catenin signaling pathway (33). High expression of CBX1 is significantly associated with a large tumor size, tumor vascular invasion, poor tumor differentiation, and prognosis in HCC patients (33, 34). High CBX1 expression was found to be significantly associated with poor prognosis in gastric cancer patients receiving adjuvant 5-fluorouracil-based chemotherapy (35). However, little is known about the role of CBX1 in ccRCC. In this study, we found that the mRNA expression of CBX1 was downregulated in ccRCC tissues compared to that in normal tissues. Low CBX1 levels are associated with advanced cancer stage and tumor grade. Moreover, the Kaplan-Meier curve showed that low expression of CBX1 was significantly associated with shorter OS and DFS. Our results are the first to indicate the anti-tumor effect of CBX1 in ccRCC, which is contrary to its pro-tumor effect in some cancers, such as liver cancer.

In 2014, Clermont et al. conducted genotranscriptomic meta-analysis and reported that the expression of CBX2 was higher in many tumors compared to that in normal tissues (39). The most represented cancer types originated from the colon, breast, stomach, and lungs (39). CBX2 overexpression and amplification are closely associated with metastatic progression and shorter OS, especially in breast cancer (40, 41). However, it has also been reported that CBX2 is expressed at similar levels in ccRCC tissues and normal tissues, and the expression of CBX2 is not associated with the prognosis of ccRCC (42). In our study, we found that CBX2 was not related to the expression, tumor characteristics, or prognosis of ccRCC, which is consistent with the results of previous research. Our results showed that CBX2 may not play a role in ccRCC.

Overexpression of CBX3 has also been observed in many malignancies such as lung cancer (43, 44), gastric cancer (36, 45), and tongue squamous cell carcinoma (46, 47). High CBX3 targets CDKN1A to promote the proliferation of U87 cells and predicts poor recurrence-free survival and OS in glioma patients (48). Moreover, CBX3 facilitates the progression of lung adenocarcinoma by directly repressing NCOR2 and ZBTB7A expression (43). However, a study has also shown that the expression of CBX3 can mediate the tumor suppression effect of lncRNA LINC00998 in malignant glioma (49). These studies suggest that CBX3 functions as both an oncogene and a tumor suppressor. In our study, CBX3 expression was increased in ccRCC, and the expression of CBX3 was correlated with advanced cancer stage and tumor grade. In addition, high CBX3 expression was significantly associated with poor OS in ccRCC patients, indicating that CBX3 is an oncogene in ccRCC.

CBX4 is the most studied member of the CBX family. CBX4 has been reported to up-regulate in multiple human tumors including lung adenocarcinoma (50), osteosarcoma (51), breast cancer (52), and cervical cancer (53). And the overexpression of CBX4 has been reported to predict shorter OS in several cancers (54, 55). In ccRCC, CBX4 interacts with HDAC1 to transcriptionally inhibit KLF6, acting as an oncogene with prognostic potential (12). In addition, CBX4 expression can mediate the pro-tumor effect of circTLK1 in RCC (56). However, it has also been reported that CBX4 suppresses cell migration, invasion, and cancer metastasis in colorectal cancer, and high expression of CBX4 is associated with better OS in CRC patients (7). In our study, the expression of CBX4 was upregulated in ccRCC tissues compared to that in normal tissues, and the expression of CBX4 was associated with advanced cancer stage and tumor grade. Moreover, high CBX4 expression was significantly associated with poor OS in ccRCC patients, suggesting that CBX4 promotes ccRCC progression.

CBX5 expression has been found in multiple cancers including breast cancer (57), gastric cancer (58), and lung cancer (59). Upregulation of CBX5 is associated with increased cell proliferation and poor clinical prognosis (59). However, in metastatic colon cancer, thyroid carcinoma, and breast cancer cells, CBX5 expression was found to be downregulated compared to that in poorly invasive or non-metastatic cells (57). Therefore, CBX5 has been characterized as a suppressor of metastasis. In our study, the expression of CBX5 was downregulated in ccRCC tissues compared to that in normal tissues. Low CBX5 levels were associated with advanced cancer stage and tumor grade. In addition, the survival analysis curve showed that low expression of CBX5 was significantly associated with shorter OS in ccRCC patients. Our results revealed the anti-tumor effect of CBX5 in ccRCC.

CBX6 and CBX7 have been found to play contradictory roles in human cancers. The present study suggested that CBX6 and CBX7 could function as both oncogenes and tumor suppressors, depending on the tumor type and cellular context. For example, high CBX6 expression promotes cell growth by regulating the S100A9/NF-κB/MAPK pathway in hepatocellular carcinoma (60). Meanwhile, CBX6 suppressed the progression of breast cancer by significantly downregulating bone marrow stromal cell antigen-2 (61). Similarly, CBX7 acts as a tumor suppressor in several cancers including thyroid cancer (8), colorectal cancer (62). In contrast, in prostate cancer and ovarian cancer, CBX7 is upregulated and acts as a tumor promoter (63, 64). In our study, CBX6 and CBX7 were favorable prognostic factors for ccRCC, and low CBX6 and CBX7 expression was positively associated with advanced cancer stage and tumor grade in ccRCC patients. Our results are consistent with the tumor suppression effects of CBX6 and CBX7.

Cumulative studies have shown that CBX8 can serve as a therapeutic target and a valuable prognostic marker for multiple cancers. In hepatocellular carcinoma, CBX8 upregulates EGR1 and miR-365-3p to stimulate the AKT/β-catenin pathway and shows oncogenic activity (65). Overexpression of CBX8 induces cell proliferation but inhibits cell migration, invasion, and metastasis in esophageal squamous cell carcinoma by repressing Snail (66). Moreover, CBX8 promotes cell proliferation by repressing the p53 pathway and serves as a predictor for muscle-invasive bladder cancer (67). In our study, CBX8 was expressed at similar levels in ccRCC tissues and normal kidney tissues, and CBX8 expression was not associated with advanced tumor characteristics. The survival analysis curve showed that CBX8 expression was an unfavorable prognostic factor for patients with ccRCC.

Several studies have suggested that tumor-infiltrating immune cells can influence tumor progression and recurrence and are closely related to the response to immunotherapy and clinical outcomes of cancer patients (68, 69). In our study, we found a significant relationship between the expression of CBXs and the infiltration of immune cells (B cells, CD8+ T cells, CD4+ T cells, macrophages, neutrophils, and dendritic cells), indicating that CBXs might influence the immune status of ccRCC patients.

## Conclusions

In conclusion, we firstly systematically investigated the expression and prognostic value of CBXs in ccRCC using an online database. Our results indicated that increased expression of CBX3 and CBX4 promoted ccRCC progression, while CBX1, CBX5, CBX6, and CBX7 were favorable factors in ccRCC. Although more studies should be performed to validate our results, our work provides new insights for selecting therapeutic targets and appropriate drugs for ccRCC patients, and our findings will help determine more accurate predictors for the survival of patients with ccRCC.

## Data Availability Statement

The original contributions presented in the study are included in the article/**Supplementary Material**. Further inquiries can be directed to the corresponding author.

## Ethics Statement

The studies involving human were reviewed and approved by the ethics committee of Tong Xu First Hospital. The patients/participants provided their written informed consent to participate in this study.

## Author Contributions

YZ and FP had the idea and wrote the article. ZP and ZL performed the literature search and data analysis. YL and NL drafted and critically revised the work. All authors contributed to the article and approved the submitted version.

## Funding

This work was supported by National Natural Science Foundation of China (81602572, 81873574), Natural Science Foundation of Hunan Province, China (2020JJ4905) and Scientific Research Project of Hunan Provincial Health Commission, China (20200489).

## Conflict of Interest

The authors declare that the research was conducted in the absence of any commercial or financial relationships that could be construed as a potential conflict of interest.

## Publisher’s Note

All claims expressed in this article are solely those of the authors and do not necessarily represent those of their affiliated organizations, or those of the publisher, the editors and the reviewers. Any product that may be evaluated in this article, or claim that may be made by its manufacturer, is not guaranteed or endorsed by the publisher.

## Supplementary Material

Supplementary Figure 1 GSEA result of CBXs mRNA high subgroup *versus* low subgroup (GSEA).
