# Supplementary figures and images for: Comprehensive Analysis of the Expression and Prognosis Value of Chromobox Family Members in Clear Cell Renal Cell Carcinoma
Source: Front Oncol. 2021 Jul 28;11:700528. doi: 10.3389/fonc.2021.700528 (PMC8357267; doi:10.3389/fonc.2021.700528)

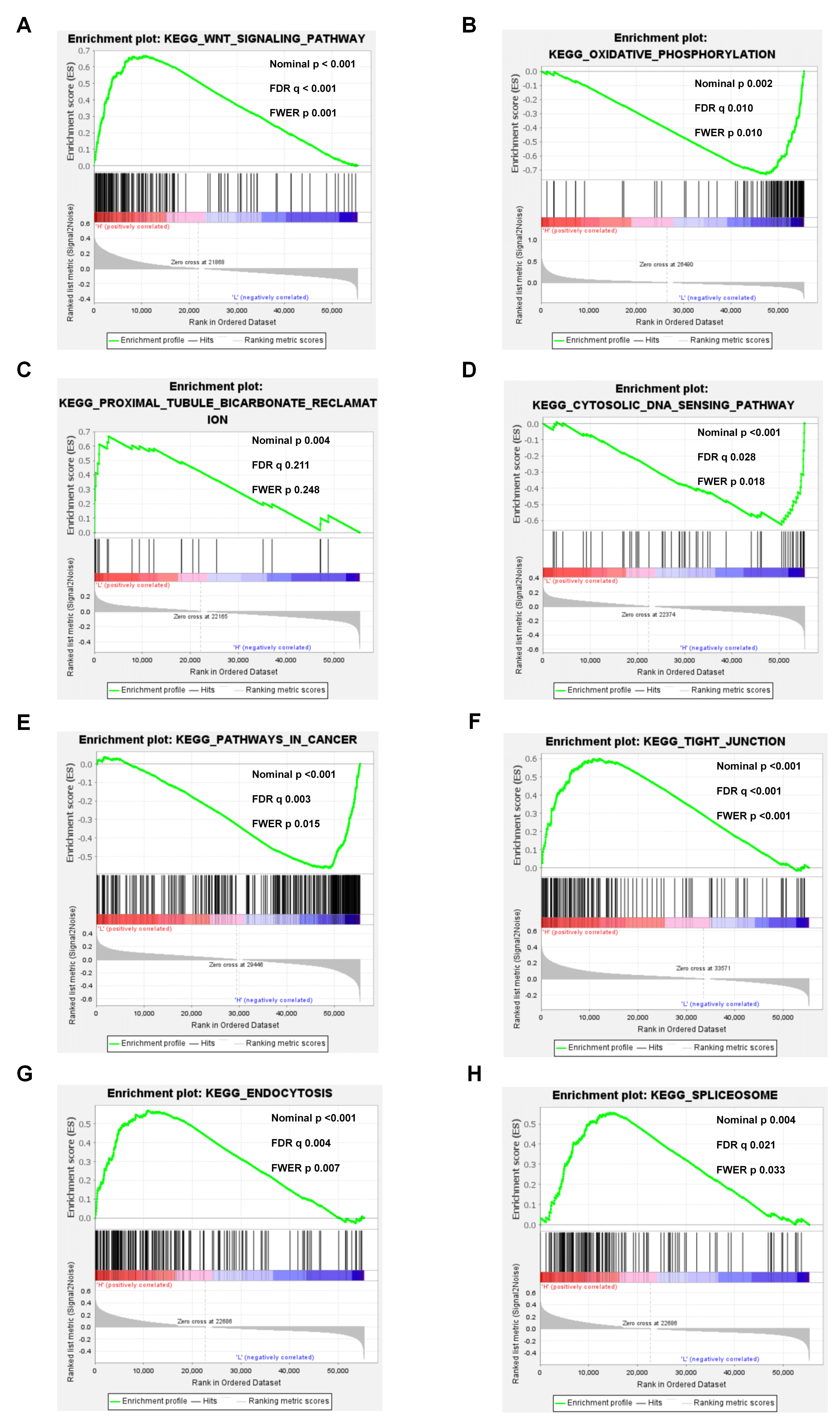

Supplement: Supplementary Figure 1 — GSEA result of CBXs mRNA high subgroup versus low subgroup (GSEA). [file Image_1.tif]
